# Oxymatrine ameliorates experimental autoimmune encephalomyelitis by rebalancing the homeostasis of gut microbiota and reducing blood-brain barrier disruption

**DOI:** 10.3389/fcimb.2022.1095053

**Published:** 2023-01-12

**Authors:** Ming-Liang Zhang, Wei-Xia Li, Xiao-Yan Wang, Ya-Li Wu, Xiao-Fei Chen, Hui Zhang, Liu-Qing Yang, Cheng-Zhao Wu, Shu-Qi Zhang, Yu-Long Chen, Ke-Ran Feng, Bin Wang, Lu Niu, De-Xin Kong, Jin-Fa Tang

**Affiliations:** ^1^ Department of Pharmacy, the First Affiliated Hospital of Henan University of Chinese Medicine, Zhengzhou, China; ^2^ Henan Province Engineering Research Center of Clinical Application, Evaluation and Transformation of Traditional Chinese Medicine, Henan Provincial Key Laboratory for Clinical Pharmacy of Traditional Chinese Medicine, Zhengzhou, China; ^3^ Chengdu University of Chinese Medicine, Chengdu, China; ^4^ School of Pharmacy, Henan University of Chinese Medicine, Zhengzhou, China

**Keywords:** oxymatrine, experimental autoimmune encephalomyelitis, gut microbiota, brain-gut axis, short-chain fatty acids

## Abstract

**Background:**

Increasing evidence suggests that gut dysbiosis can directly or indirectly affect the immune system through the brain-gut axis and play a role in the occurrence and development of Multiple sclerosis (MS). Oxymatrine (OMAT) has been shown to ameliorate the symptoms of MS in the classical experimental autoimmune encephalomyelitis (EAE) model of MS, but whether its therapeutic role is through the correction of gut dysbiosis, is unclear.

**Methods:**

The effects of OMAT on intestinal flora and short-chain fatty acids in EAE model mice were evaluated by 16S rRNA sequencing and GC-MS/MS, respectively, and the function change of the blood-brain barrier and intestinal epithelial barrier was further tested by immunohistochemical staining, Evans Blue leakage detection, and RT-qPCR.

**Results:**

The alpha and beta diversity in the feces of EAE mice were significantly different from that of the control group but recovered substantially after OMAT treatment. Besides, the OMAT treatment significantly affected the gut functional profiling and the abundance of genes associated with energy metabolism, amino acid metabolism, the immune system, infectious diseases, and the nervous system. OMAT also decreased the levels of isobutyric acid and isovaleric acid in EAE mice, which are significantly related to the abundance of certain gut microbes and were consistent with the reduced expression of TNF-a, IL-6, and IL-1b. Furthermore, OMAT treatment significantly increased the expression of ZO-1 and occludin in the brains and colons of EAE mice and decreased blood-brain barrier permeability.

**Conclusion:**

OMAT may alleviate the clinical and pathological symptoms of MS by correcting dysbiosis, restoring gut ecological and functional microenvironment, and inhibiting immune cell-mediated inflammation to remodel the brain-gut axis.

## 1 Introduction

Multiple sclerosis (MS) as chronic neuroinflammation and demyelinating disease in the central nervous system (CNS), is associated with key pathological features: multifocal inflammation, disseminated demyelination, axonal loss, and neurological disability (Thompson et al., 2018; Xu et al., 2020). For the treatment of MS, recent studies have found that oral probiotics can significantly ameliorate the incidence of MS (Tankou et al., 2018), while transplantation of fecal bacteria from MS patients can aggravate the incidence of its classical animal model (experimental autoimmune encephalomyelitis, EAE) mice (Berer et al., 2017; Cekanaviciute et al., 2017), suggesting therapeutic targeting of the gut microbiota as a treatment for MS (Leprun and Clarke, 2019), which aroused great interest in the study of the brain-gut axis. With the growing evidence of gut microbiota disorder in MS patients, the mechanism of gut microbiota disorder exacerbating MS is gradually clear. The current main view is that the disordered gut microbiota mediates the inflammatory immune response of CNS by changing the permeability of the intestinal epithelial barrier (IEB) and blood-brain barrier (BBB) and affecting the expression of neurotransmitters related to the brain-gut axis in MS patients or EAE mice (Preziosi et al., 2013; Chu et al., 2018; Stanisavljevic et al., 2018). Therefore, researching and developing gut microbiota-modifying therapies, such as drugs with antibacterial and immunomodulatory effects may be extremely urgent for the treatment of MS.

Significant progress has been made in therapeutic strategies for MS, including cladribine, dimethyl fumarate, fingolimod, teriflunomide, alemtuzumab, and ocrelizumab. However, long-term use of MS medications is costly and may cause certain side effects, such as liver injury, lymphopenia, and infections (Antonazzo et al., 2019; Moiola et al., 2020). Importantly, recent studies have found that some types of traditional herbal medicine can ameliorate clinical severity and reduce the frequency of clinical exacerbations in relapsing MS with fewer side effects and minor financial burdens (Kou et al., 2014; Zhou and Fan, 2015; Dou et al., 2021; Zha et al., 2021). Therefore, natural products may be a promising treatment for MS.

*Sophora flavescens* Ait. (Leguminosae), as traditional herb medicine, contains an important component namely oxymatrine (OMAT), which has been reported to exhibit significant anti-tumor, anti-virus, hepatoprotective, and immunomodulatory effects (Rashid et al., 2019; Zhang et al., 2020; Li et al., 2021). OMAT has been tested in a clinical trial for the treatment of human hepatitis B, with significant therapeutic effects and without noticeable side effects (Zhang et al., 2016). Our previous studies have found that OMAT could delay the development of the EAE and modulate immune responses (Liu et al., 2014; Zhang et al., 2017). It is reported in the literature that OMAT has a certain regulatory effect on the gut microbiota and intestinal barrier (Li et al., 2019; Wu et al., 2021), whether it treats MS by remodeling gut microbiota homeostasis and IEB function is not clear. Herein, we investigated the mechanisms of OMAT on the function of the IEB and BBB, and the regulating effect on intestinal microecology in the EAE mice.

## 2 Materials and methods

### 2.1 Animals and EAE induction

C57BL/6 female mice, 8-10 weeks of age (18-20 g), were obtained from SPF Biotechnology Co., Ltd. (Beijing, China), and housed in specific pathogen-free conditions at the Fifth Medical Center of PLA General Hospital (animal ethics committee approval No. YFYDW2020017), Beijing, China. All efforts were made to reduce animal suffering, and the Institutional Committee on Care and Use of Research animals approved the procedures used in this study. EAE was induced as described previously (Dou et al., 2021). Inactivated Mycobacterium tuberculosis (#231141, Difco) was added with Freund’s incomplete adjuvant (#F5506, Sigma) to prepare a 10 mg/mL solution, which was fully mixed with an equal volume of myelin oligodendrocyte glycoprotein peptide (MOG35-55, MEVGWYRSPFS RVVHLYRNGK; GenScript) solution (2 mg/mL) dissolved in sterile phosphate-buffered saline (PBS) to prepare water in oil antigen emulsion. Mice anesthetized with pentobarbital at four points on both sides of the midline of the head and back, a total of 0.2 mL/mouse. On 0 h and 48 h post-immunization (p.i.), 200 ng/mouse pertussis toxin (#180, List Biological) was injected intraperitoneally.

### 2.2 MAT treatment and clinical evaluation

Immunized mice were randomly divided into two groups (n=9 each group), and starting from day 11 p.i., 40 mg/kg/day OMAT (Macklin Biochemical Co., Ltd, Shanghai, China) or equal volumes of normal saline were administered in immunized mice by oral served as the EAE_OMAT group or EAE group, respectively. Non-immunized naive mice (n=9 in each group) received the same volumes of normal saline or OMAT served as the CON group or CON_OMAT group, respectively.

The mice were monitored and weighed daily by two independent observers to evaluate the clinical scores of EAE. Neurological assessments were recorded with a five-point standardized rating scale (Dou et al., 2021): 0, no deficit; 1, tail paralysis; 2, incomplete hind limb paralysis; 3, complete hind limb paralysis; 4, complete hind limb paralysis and partial forelimb paralysis; 5, moribund state or death.

### 2.3 Histopathological evaluation

On day 20 post-immunization (p.i.), mice were sacrificed, colons and spinal cords were harvested after extensive perfusion with saline, embedded in paraffin, and processed for histological evaluation, and fecal samples were collected in a sterile operating table in a sterile cryopreservation tube and placed in a refrigerator at -80°C for standby. Inflammatory infiltration in lumbar enlargement of spinal cords and colons was determined by Hematoxylin and Eosin (HE) staining, and demyelination in lumbar enlargement of spinal cords was determined by Luxol Fast Blue (LFB) staining.

### 2.4 Evans Blue leakage detection

The BBB permeability was determined by Evans Blue (EB) dye (Jiao-Yan et al., 2021). Mice were given 2% EB (2 mL/kg) *via* tail vein injection 90 minutes before being sacrificed. After weighing the brain, 50% trichloroacetic acid was added in the ratio of 1:3 (W/V) to make homogenate and centrifuged (12000 g. 15 minutes). The supernatant was collected, and its absorbance at 620 nm was measured by a microporous plate spectrophotometer to calculate EB transmittance.

### 2.5 Microbiota sequencing

Total bacterial DNA was extracted from fresh stool samples by the E.Z.N.A.^®^ Stool DNA Kit (Omega Bio-Tek, U.S). The DNA in stools was quantified through agarose gel electrophoresis and amplified V3-V4 variable regions of the 16S rRNA gene with specific primers (338F: 5’-ACTCCTACGGGAGGCAGCAG-3’ and 806R: 5’-GGACTACHVGGGTWTCTAAT-3’). To evaluate alpha-diversity, the levels of OTUs for each group. and the alpha diversity indices, including Ace, Chao, Coverage, Shannon, Simpson, and sobs index, are usually used for evaluating the community richness and community diversity. To analyze beta-diversity, principal coordinates analysis (PCA) analysis which is a phylogenic tree-based metric was used (Calderón et al., 2017). Phylogenetic Investigation of Communities by Reconstruction of Unobserved States (PICRUSt) was further used for genome prediction of microbial communities in this study.

### 2.6 Quantification of fecal short-chain fatty acids

SCFAs, including acetic acid (AA), propionic acid (PA), isobutyric acid (IBA), butyric acid (BA), isovaleric acid (IVA), valeric acid (VA), and hexanoic acid (HA), were extracted and analyzed by gas chromatography-mass spectrometry/mass spectrometry (GC-MS/MS) according to the protocol described in Margareta Nyman (Zhao et al., 2006). Accurately weigh the sample, add 0.5% phosphoric acid solution, grind it evenly by ball mill, vortex and mix it evenly, ultrasonic in the ice bath for 5 minutes, 4°C, 12000 rpm, centrifuge for 10 minutes, and take 100 μL supernatant and 500 μL methyl tert-butyl ether (MTBE) solvent containing internal standard, vortex for 3 minutes, ultrasonic for 5 minutes under ice bath, centrifuge for 10 minutes at 12000 rpm at 4°C, take the supernatant and store it in the refrigerator at - 80°C for standby. The supernatant was collected and used for GC-MS/MS analysis. Agilent 7890B-7000D was employed for GC-MS/MS analysis of SCFAs.

### 2.7 Immunohistochemistry analysis

On day 20 p.i., mice were deeply anesthetized with 2% pentobarbital sodium and extensively perfused by normal saline. Then lumbar spinal cords, brain, and colon were immediately removed and fixed in 4% paraformaldehyde and serial cryostat longitudinal sections were cut at a 5 µm thickness for immunohistochemistry analysis. After deparaffinization in xylol, sections were transferred to gradient ethanol and then incubated with 3% H_2_0_2_ for 30 minutes at room temperature. After washing in PBS, non-specific binding was blocked with bovine serum for 30 minutes at 37°C and incubated with anti-occludin, anti-zonula occludens-1 (anti-ZO-1) (Santa Cruz Biotechnology, Dallas, TX, USA) at 4°C overnight. Sections were washed again and then incubated with corresponding secondary antibodies at 37°C for 30 minutes. The chromophore product was developed using Horseradish Peroxidase-Streptavidin (HRP-Streptavidin) and 3,3N-Diaminobenzidine Tertrahydrochloride (DAB) (Beijing Zhongsan Biotech Co, LTD, Beijing, China). Positive cells in a restricted area were determined to represent target protein expression.

### 2.8 Quantitative reverse transcription-polymerase chain reaction

The cervical parts of the spinal cord and colon were harvested and flash-frozen in liquid N2 on day 20 p.i., and then stored at -80°C before use. Total RNA was isolated by RNA-Quick Purification Kit according to the manufacturer’s instructions (ES Science, RN001, China), and cDNA was synthesized using reverse transcriptase (RT) by Fast All-in-One RT Kit (ES Science, RT001, China) followed by RT-qPCR (Thermal Cycler Device Real-Time System, Takara) using 2×Super SYBR Green qPCR Master Mix (ES Science, QP002, China) with appropriate primers and probe (Table 1). The cycle threshold (Ct) values of TNF-α, IL-6, IL-1β, ZO-1, and occludin were obtained and normalized to that of GAPDH (all reagents from TIANYI HUIYUAN, Beijing, China). Based on the expression of target genes normalized to GAPDH, we calculated and presented the relative quantification to GAPDH as fold change compared to the control. The above gene sequence information can be queried in **Supplementary Table 1** (SI Appendix, **Table S1**).

### 2.9 Statistical analysis

All statistical analyses were conducted with R version 3.3.1 or GraphPad Prism 8.0 (Inc., La Jolla, CA, USA). Significant differences were evaluated using the two-tailed Student’s t-test or Wilcoxon test, except that multiple treatment groups were compared within individual experiments by the Wilcoxon rank-sum test or LSD-t test. The relevance between the abundance of microbial genera/OTUs and neurologic score or the levels SFCAs was performed by using spearman’s rank correlation coefficient. The ranking regression of environmental factors was analyzed according to beta diversity. All data were presented as mean ± SD. *P<0.05* was considered significant.

## 3 Results

### 3.1 OMAT treatment ameliorates the clinical signs of ongoing EAE

**Figure 1:**
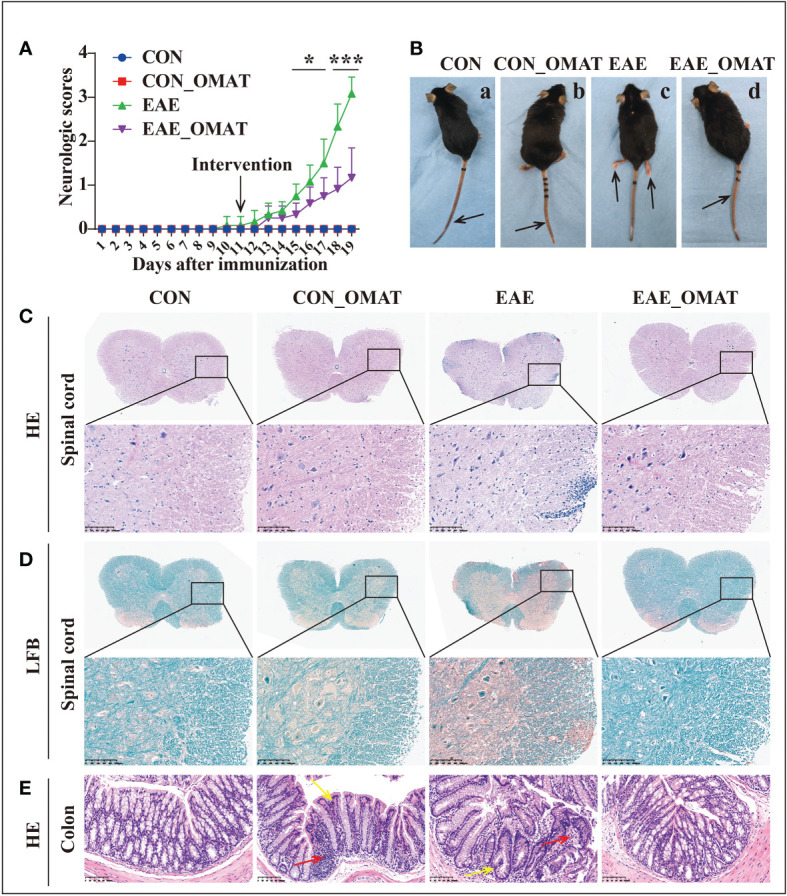
Results of the pathological and histological examination of the spinal cord and colon of the mice from the four groups (n = 6 in each group). **(A)** Clinical scores and **(B)** clinical phenotypes of mice from the different groups. The black arrow indicates the paralyzed area. a, CON group mice with no deficit; b, CON_EAE group mice with no deficit; c, EAE group mice showing paralysis of both hind limbs; d, EAE_OMAT group mice showing tail paralysis. **(C, D)** Representative images of the histology of the spinal cord were determined by HE **(C)** and LFB **(D)** staining. Original magnification, 4X; the partial enlarged picture of each group was magnified 20X. **(E)** Representative images of the histology of the colon determined by HE staining (20X). Red arrows indicate colon tissue infiltration by inflammatory cells, yellow arrows indicate the change in crypt structure. Data are expressed as the mean ± SD and statistical differences are represented by *, P < 0.05; ***, P < 0.001 based on Wilcoxon rank-sum test.

As shown in **Figure 1A**, EAE group mice had the first signs of EAE on day 11 p.i., EAE_OMAT group mice showed a significant decrease in the severity of EAE from the 15th-day p.i. as compared with EAE group mice (all *P<0.05*). On day 20 p.i., EAE group mice were observed unilateral hind limb paralysis or bilateral hind limb paralysis, while EAE_OMAT group mice were observed tail paralysis or incomplete hind limb paralysis (**Figure 1B**). Consistent with clinical scores, the histopathological analysis revealed less inflammation (**Figure 1C**) and demyelination (**Figure 1D**) in the lumbar spinal cords samples from EAE_OMAT group mice. Furthermore, histopathology revealed milder crypt structure change and less inflammatory infiltration in the colons of CON_OMAT and EAE group mice, the inflammatory and crypt structure change in the OMAT-treated EAE mice has been distinctly ameliorated than EAE group mice (**Figure 1E**).

### 3.2 OMAT administration altered the alpha- and beta-diversities of the gut microbiome in EAE mice

**Figure 2:**
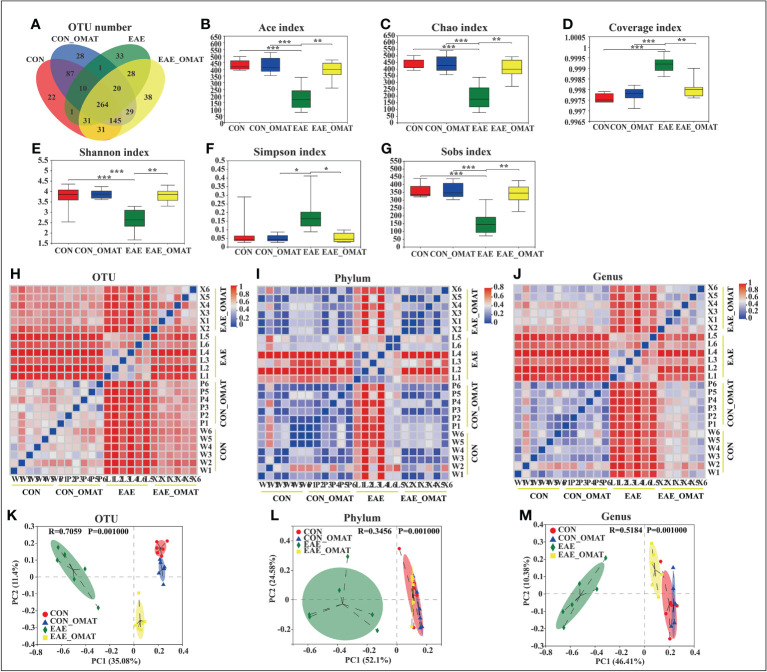
Gut microbiota alpha- and beta-diversities in the four mice groups (n = 6 in each group). **(A–D)** Alpha-diversity indices. **(A)** The number of species and **(B)** Ace-estimated OTUs, **(C)** the Chao index value **(D)** the Coverage index value **(E)** the Shannon index value **(F)** the Simpson index value, and **(G)** the Sobs index value of fecal microbiota from the four groups based on 16S rRNA analysis. Each box plot represents the median, interquartile range, minimum, and maximum values. **(H–M)** Beta-diversity indices. Sample-distance heatmap based on weighted UniFrac analysis at the level of OTUs **(H)**, phylum **(I)**, and genus **(J)**. Principal coordinates analysis (PCoA) was used to study the similarity or difference of the microbial community composition at the level of OTUs **(K)**, phylum **(L)**, and genus **(M)**. Data are expressed as the mean ± SD and statistical differences are represented by *, P < 0.05; **, P < 0.01; ***, P < 0.001 based on the Wilcoxon rank-sum test with the Benjamini–Hochberg method for multiple group comparisons.

From the Venn diagram in OUT levels (**Figure 2A**), 591 species in the CON group, 584 species in the CON_OMAT group, 388 species in the EAE group, and 586 species in the EAE_OMAT group were observed. The alpha diversity indices, including Ace, Chao, Coverage, Shannon, Simpson, and Sobs index, are usually used for evaluating community richness and community diversity (**Figures 2B–G**). In this study, Ace, Chao, Shannon, and sobs values in the EAE_OMAT-treated mice were markedly higher than that in the EAE group (all *P<0.05*), while coverage and Simpson values in the EAE_OMAT-treated mice were markedly lower than that in the EAE group (all *P<0.05*). In addition, beta-diversity in the four groups was evaluated by Bray-Curtis analysis (Heatmap diagram) and Principal coordinates analysis (PCoA) using the 16S data. According to the distance and separation of each sample in each group in **Figures 2H–M**, it is evident that the CON, CON_OMAT, and EAE_OMAT are completely separated from the EAE group in OUT (**Figures 2H, K**), phylum (**Figures 2I, L**) and genus (**Figures 2J, M**) levels, and there are differences between the EAE_OMAT group and the CON group in genus and OUT levels (all *P<0.05*).

### 3.3 OMAT treatment influenced the gut microbial abundance and phenotypes in EAE mice

**Figure 3:**
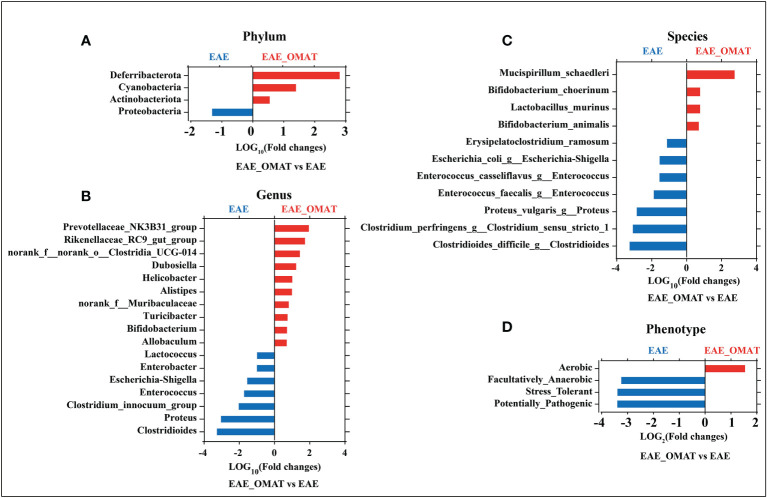
Effect of oxymatrine treatment on the gut microbial abundance and microbiome phenotypes in EAE mice. The fold-change (LOG_2_ or LOG_10_) of relative abundance at the level of phylum **(A)**, genus **(B)**, species and **(C)**, phenotype **(D)** between the EAE group and EAE_OMAT groups (*P < 0.05*). The Z-score based on the abundance of each KEGG ortholog is depicted from lowest (blue) to highest (red) according to the scale shown at the top. The P value was determined based on the Wilcoxon rank-sum test with the Benjamini–Hochberg method for multiple group comparisons.

Microbial abundance at different taxonomic levels was further analyzed. The phylum-level assignment identified 10 phyla with an average relative abundance of ≥0.1% in at least one of the four groups. The comparison between all pairs of the four groups identified a total of 6 distinct phyla having significant changes in abundance between any two groups (SI Appendix, Table S2), and identified the abundance of Deferribacterota, Cyanobacteria, and Actinobacteriota significantly increased and Proteobacteria significantly decreased after OMAT-treated in EAE mice (all *P<0.05*) (**Figure 3A**).

The genus-level assignment identified 42 genera with an average relative abundance of ≥0.5% in at least one of the four groups, accounting for 93.03% of the total abundance. The comparison between all pairs of the four groups identified a total of 30 distinct genera having significant changes in abundance between any two groups (SI Appendix, Table S3). Sorted by fold changes (FCs, |LOG_10_ (FCs)|>1) value and P-value (*P<0.05*), the abundance of Alistipes, Helicobacter, Dubosiella, norank_f:norank_o:Clostridia_UCG-014, Rikenellaceae_RC9_gut_group, Prevotellaceae_NK3B31_group were significantly higher in EAE_OMAT mice than EAE mice (all *P<0.05*), while the abundance of Clostridioides, Proteus, Clostridium_innocuum_group, Enterococcus, Escherichia-Shigella, Enterobacter, Lactococcus were significantly lower in EAE_OMAT group than in EAE group (**Figure 3B**).

To explore the possible association of microbial taxa with each patient group at the species level, the abundance of 38 species-equivalent clusters with an average relative abundance of ≥0.1% in at least one of the four groups were further compared, accounting for 17.79% (CON group), 19.40% (CON_OMAT group), 68.30% (EAE group), 31.88% (EAE_OMAT group) of the total abundance, respectively. The comparison between all pairs of the four groups identified a total of 28 distinct species having significant changes in abundance between any two groups (SI Appendix, Table S4). Sorted by fold changes (FCs, |LOG_10_ (FCs)|>1) value and P-value (*P<0.05*), the abundance of Mucispirillum_schaedleri was significantly higher in the EAE_OMAT group than in the EAE group (all *P<0.05*), while the abundance of Clostridioides_difficile_g:Clostridioides, Clostridium_perfringens_g:Clostridium_sensu_stricto_1, Proteus_vulgaris_g:Proteus, Enterococcus_faecalis_g:Enterococcus, Enterococcus_casseliflavus_g:Enterococcus, Escherichia_coli_g:Escherichia-Shigella, Erysipelatoclostridium_ramosum were significantly lower in EAE_OMAT group than in EAE group (**Figure 3C**).

Considering various taxonomic levels of gut microflora have changed, the corresponding microflora phenotype will also change. The study identified 9 phenotypes in at least one of the four groups including 6 distinct phenotypes having significant changes in proportion between any two groups by BugBase analysis according to the data obtained from sequencing of 16S analysis (SI Appendix, Table S5). To investigate the therapeutic effect of OMAT on EAE mice, we focused on the functional difference between the EAE group and the EAE_OMAT group. The results indicated the proportion of Facultatively_Anaerobic, Stress_Tolerant, and Potentially_Pathogenic were significantly decreased after OMAT-treated in EAE mice (all *P<0.05*), while the proportion of Aerobic was significantly increased after OMAT-treated in EAE mice (*P<0.05*) (**Figure 3D**).

### 3.4 Association between significantly increased or decreased microbial genera/OTUs in EAE-related groups and neurologic function scores

**Figure 4:**
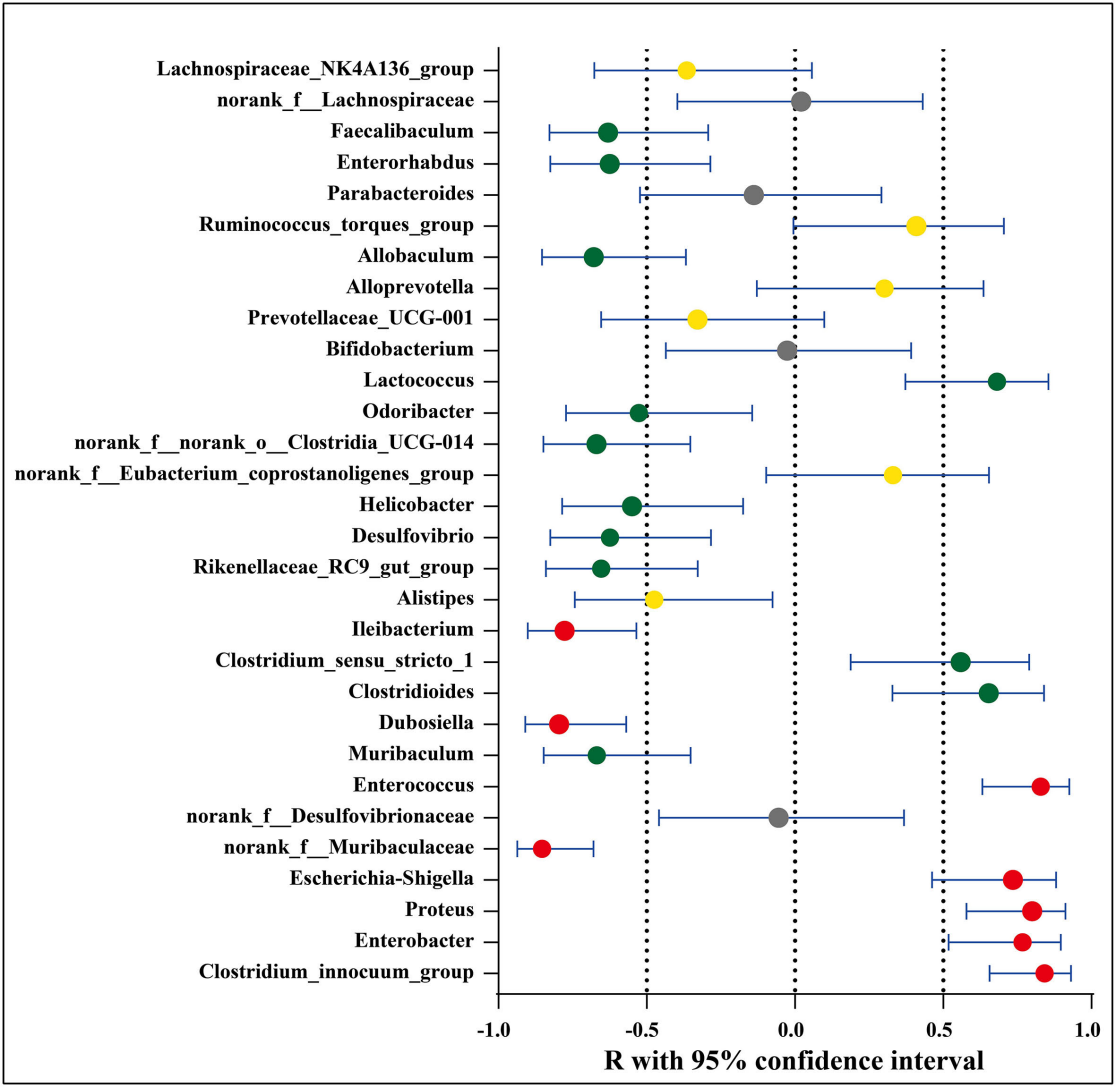
Association between significantly increased or decreased microbial genera/OTUs in EAE groups and corresponding neurologic function scores. Red dots represent a strong correlation (0.7 ≤ R < 1), green dots represent a moderate correlation (0.5 ≤ R < 0.7), yellow dots represent a weak correlation (0.3 ≤ R < 0.5), and gray dots represent a poor correlation (0 < R < 0.3). The relation between the abundance of microbial genera/OTUs and the neurologic function scores were performed using Spearman’s rank correlation coefficient.

The possible association between the microbial species and disease activity (neurologic function scores) was further analyzed by comparing the abundance of the 30 genera/OTUs which existed significant differences between the EAE group and EAE_OMAT group. The linear relationship strength and direction are classified according to the correlation coefficient R-value (Rumsey, 2016). As **Figure 4** showed, Clostridium_innocuum_group, Enterococcus, Proteus, Enterobacter, and Escherichia-Shigella had strong uphill linear relationships in abundance between the neurological function score and EAE-related samples (R=0.84, 0.83, 0.80, 0.77, 0.74, respectively), while Ileibacterium, Dubosiella, norank_f:Muribaculaceae had strong downhill linear relationships in abundance between the neurological function score and EAE-related samples (R=-0.78, -0.80, -0.85, respectively). Meanwhile, the abundance of Lactococcus, Clostridioides, Clostridium_sensu_stricto_1 and disease severity correlated positively (R=0.68, 0.65, 0.56, respectively), and Odoribacter, Helicobacter, Desulfovibrio, Enterorhabdus, Faecalibaculum, Rikenellaceae_RC9_gut_group, Muribaculum, norank_f:norank_o:Clostridia_UCG-014, Allobaculum and disease severity were negatively related to (R=-0.53, -0.55, -0.62, -0.63, -0.63, -0.65, -0.67, -0.67, -0.68, respectively). (SI Appendix, Table S6).

### 3.5 OMAT altered the functional profiling of the gut microbiomes

**Figure 5:**
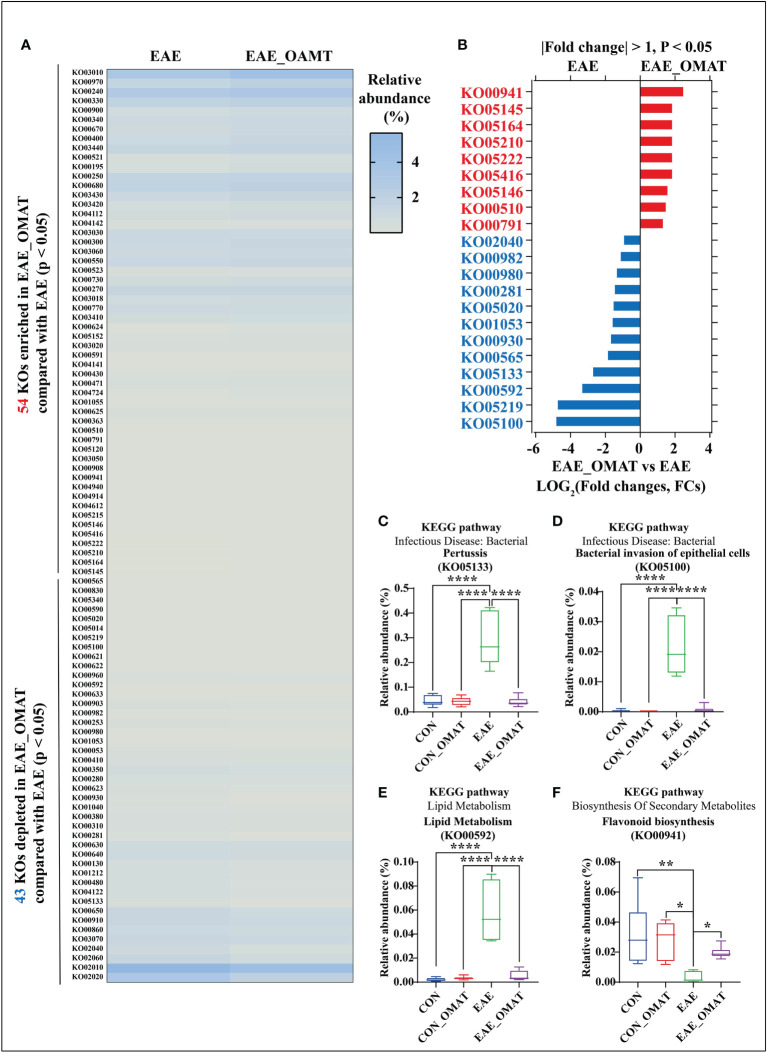
Effect of oxymatrine on the functional profiling of the gut microbiome. **(A)** The list of 54 significantly enriched and 43 significantly depleted KEGG ortholog (KOs) in the EAE_OMAT group than in the EAE group (*P < 0.05*). **(B)** Ranked based on the fold changes of relative abundance (|LOG_2_(FCs)| ≥ 1), these individual pathways include at least one of the 9 significantly enriched KOs (EAE_OMAT-enriched KOs) or one of the 12 significantly depleted KOs (EAE_OMAT-depleted KOs) in the comparisons between the EAE and EAE_OMAT groups. **(C–F)** Relative abundance of the 4 significantly enriched or depleted KOs in EAE compared with EAE_OMAT. The relative abundance of pertussis (KO05133; **(C)**, bacterial invasion of epithelial cells (KO05100; **(D)**, alpha-Linolenic acid metabolism (KO00592; **(E)**, flavonoid biosynthesis (KO00941; **(F)** between the four subject groups. Data are expressed as the mean ± SD and statistical differences are represented by *, *P* < 0.05, **, *P* < 0.01, and ****, *P* < 0.0001 based on Wilcoxon rank-sum test with the Benjamini–Hochberg method for multiple group comparisons.

Based on the Kyoto Encyclopedia of Genes and Genomes (KEGG) database (Kanehisa et al., 2019), a total of 201 KEGG orthologs (KOs) in the four subject groups were identified by PICRUSt (SI Appendix, Table S7). Of them, 1 had significant changes in abundance between the CON group and CON_OMAT group, 95 had significant changes in abundance between the CON group and the EAE group, and 97 had significant changes in abundance between the EAE group and EAE_OMAT group. In the comparison of EAE and EAE_OMAT, we identified 54 significantly enriched and 43 significantly depleted KOs in EAE_OMAT with *P<0.05* by Wilcoxon test, respectively (**Figure 5A**). Sorted by P-value (*P<0.05*) and FCs value (|LOG_2_ (FCs)|>1), we picked out 21 KOs (**Figure 5B**). Among these pathways, 6 pathways including influenza A (KO05164), pertussis (KO05133), bacterial invasion of epithelial cells (KO05100), prion diseases (KO05020), viral myocarditis (KO05416), and flagellar assembly (KO02040) were involved in Bacterial or Viral Infection; 3 pathways including colorectal cancer (KO05210), small cell lung cancer (KO05222), and bladder cancer (KO05219) were involved in Cancer; 4 pathways including atrazine degradation (KO00791), drug metabolism-cytochrome P450 (KO00982), metabolism of xenobiotics by cytochrome P450 (KO00980), and caprolactam degradation (KO00930) were involved in Xenobiotics Biodegradation and Metabolism; 2 pathways including ether lipid metabolism (KO00565), and Alpha-Linolenic Acid Metabolism (KO00592) were involved in Lipid Metabolism; 2 pathways including geraniol degradation (KO00281), and biosynthesis of siderophore group nonribosomal peptides (KO01053) were involved in Metabolism Of Terpenoids and Polyketides; 2 pathways including toxoplasmosis (KO05145), and amoebiasis (KO05146) were involved in Parasitic Infection; 1 pathways including N-Glycan biosynthesis (KO00510) was involved in Glycan Biosynthesis and Metabolism; 1 pathways including flavonoid biosynthesis (KO00941) was involved in Biosynthesis Of Other Secondary Metabolites. The study only showed the four metabolic pathways with large changes in relative abundances, such as pertussis (**Figure 5C**), bacterial invasion of epithelial cells (**Figure 5D**), α- Linolenic acid metabolism (**Figure 5E**), flavonoid biosynthesis (**Figure 5F**).

### 3.6 OMAT rebalanced the relative abundance of three major nutrients, the nervous system, immune system, and infectious diseases based on KEGG analysis

**Figure 6:**
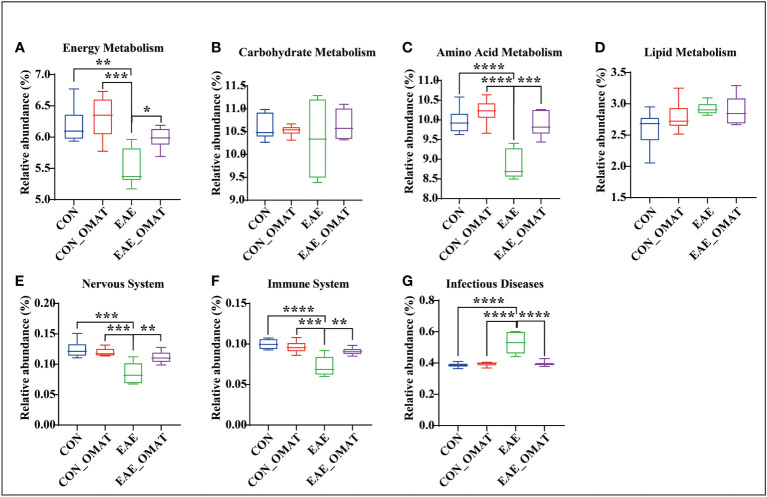
Comparison of the relative abundance of energy metabolism, nervous system, immune system, and infectious diseases based on KEGG analysis. Relative abundance of the KEGG orthologs belonging to Energy Metabolism **(A)**, Carbohydrate Metabolism **(B)**, Amino Acid Metabolism **(C)**, Lipid Metabolism **(D)**, Nervous System **(E)**, Immune System **(F)**, and Infectious Diseases **(G)** in the four mice groups. Data are expressed as the mean ± SD and statistical differences are represented by *, *P* < 0.05, **, *P* < 0.01, ***, and *P* < 0.001 based on Wilcoxon rank-sum test with the Benjamini–Hochberg method for multiple group comparisons.

Metabolism pathways related to energy metabolism (including carbohydrates, proteins, and lipids), nervous system, immune system, and infectious diseases at the KEGG subcategory level to evaluate the possible effects of OMAT on the gut microbiome were also analyzed by comparing the abundance of genes in these metabolism pathways with EAE group. The results revealed that the KEGGs for the energy metabolism, amino acid, immune system, and nervous system metabolisms were significantly increased in EAE_OMAT compared to those in EAE (all *P<0.05*) (**Figures 6A, C, E, F**), while the genes-related with infectious diseases metabolisms were significantly decreased in EAE_OMAT compared to those in EAE (all *P<0.05*) (**Figure 6G**). However, Carbohydrate Metabolism and Lipid Metabolism in EAE mice have no significant changes under OMAT intervention (**Figure 6B, D**).

### 3.7 OMAT regulates the levels of SCFAs and related gut microbiota

**Figure 7:**
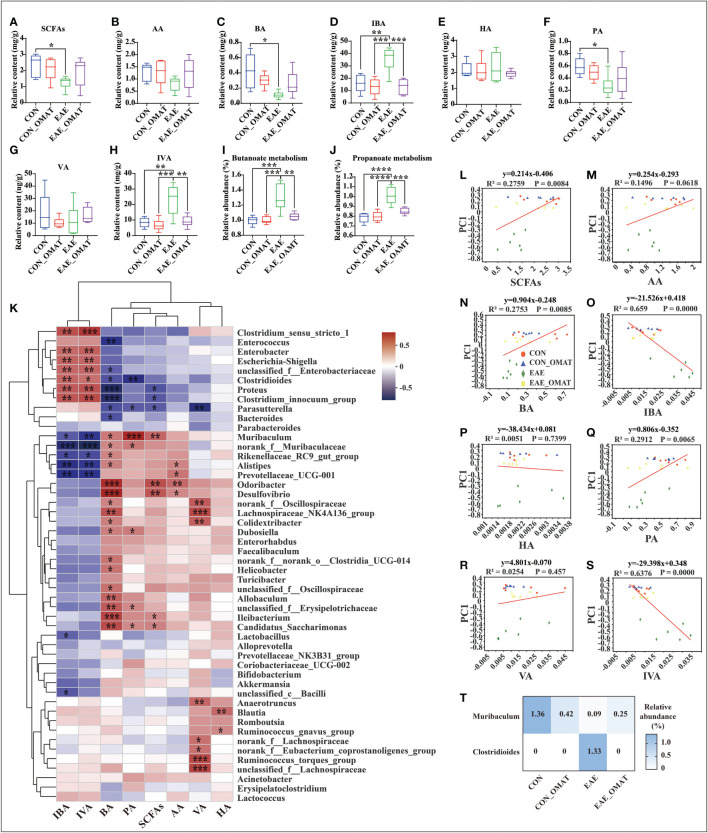
Effect of oxymatrine on the gut microbiota and the concentration of short-chain fatty acids (n = 6 in each group). **(A–I)** The concentration of SCFAs **(A)**, acetic acid (AA) **(B)**, butyrate acid (BA) **(C)**, isobutyrate acid (IBA) **(D)**, hexanoic acid (HA) **(E)**, propionic acid (PA) **(F)**, valeric acid (VA) **(G)**, and isovaleric acid (IVA) **(H)** in the feces of the four mice groups. **(I, J)** KEGG orthologs (KOs) are associated with Butyrate metabolism **(I)** and Propionate metabolism **(J)**. Correlation heatmap diagram of the concentration of short-chain fatty acids (SCFAs) and the relative abundance of gut microbes belonging to a different genus **(K)**. **(L–S)** Linear rank regression analysis of the relationship between the gut microbiome and the concentration of SCFAs **(M)**, acetic acid **(N)**, butyrate acid **(O)**, isobutyrate acid **(P)**, hexanoic acid **(Q)**, propionic acid **(R)**, valeric acid **(S)**, isovaleric acid **(T)** in the feces of the four mice groups. **(T)** Relative abundance of *Clostridioides* and *Muribaculum* in the four mice groups. The P values for **(A–H)** were derived from the LSD-t test. In **(I-J)** and **(T)**, the P value was determined based on Wilcoxon rank-sum test with the Benjamini–Hochberg method. In **(K-S)**, the relation between the abundance of microbial genera/OTUs and the concentration SFCAs was assessed using Spearman’s rank correlation coefficient. Data are expressed as the mean ± SD and statistical differences are represented by *, *P* < 0.05, **, *P* < 0.01, ***, *P* < 0.001, and ****, *P* < 0.0001.

Considering the regulatory effect of SCFAs on MS, the levels of SCFAs in fecal samples were analyzed. The result revealed were obvious potential reduction of the total levels of SCFAs in EAE group mice as compared to those from CON group mice, while this phenomenon was slightly reversed in EAE_OMAT group mice (**Figure 7A**). The levels of BA and PA were significantly lower in EAE group mice compared with CON group mice (both *P<0.05*), and the levels of IBA and IVA were significantly increased (both *P<0.05*). After OMAT treatment, the levels of IBA and IVA in EAE mice were significantly decreased (both *P<0.05*) (**Figures 7B–H**). Meanwhile, the metabolism pathways related to butyrate metabolism and propionate metabolism were significantly decreased in EAE_OMAT mice compared to EAE mice (all *P<0.05*) (**Figures 7I, J**).

The possible association between SCFAs expression and the top 50 kinds of microbial genus/OTUs according to the abundance was further analyzed by linear regression (**Figure 7K**). The results revealed the levels of total SCFAs, BA, and PA were markedly positively correlated with microbial abundance (R^2 =^ 0.2759, *P<0.01*; R^2 =^ 0.2735, *P<0.01*; R^2 =^ 0.2912, *P<0.01*; respectively), while the levels of IBA and IVA were dramatically negative correlation with microbial abundance (R^2 =^ 0.659, *P<0.0001*; R^2 =^ 0.6376, *P<0.0001*; respectively) (**Figures 7L–S**). Based on the above results, Clostridioides and Muribaculum screened out the significant correlation with BA, IBA, PA, and IVA (all *P<0.05*). The results showed the abundance of Clostridioides was found positively correlated with the expression of IBA, IVA (R=0.53; R=0.50; respectively), and negatively correlated with the expression of PA, BA (R=-0.60; R=-0.46; respectively). Meanwhile, the abundance of Muribaculum was positively correlated with the expression of PA, BA (R=0.75; R=0.46; respectively), and negatively correlated with the expression of IBA, IVA (R=-0.44; R=-0.55; respectively) (SI Appendix, Table S8). We reviewed the changes in the abundance of Clostridioides and Muribaculum in different groups and found Clostridioides was significantly reduced to the level of the normal group, and Muribaculum was increased significantly in the OMAT group while compared with the EAE group (both *P<0.05*) (**Figure 7T**).

### 3.8 OMAT down-regulates the expression of pro-inflammatory cytokines in the brains and colons of EAE mice

**Figure 8:**
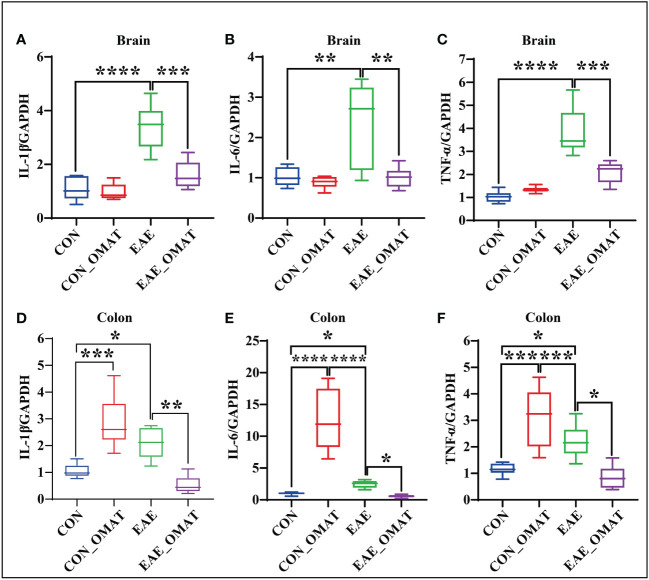
Effect of oxymatrine on the expression of pro-inflammatory cytokines in the brains and colons of EAE mice (n = 6 in each group). Results of RT-qPCR analysis showing a decrease in the mRNA expression of IL-1β **(A, D)**, IL-6 **(B, E)**, and TNF-α **(C, F)** in brain and colon samples of EAE mice after OMAT treatment. Data are expressed as the mean ± SD and statistical differences are represented by *, *P* < 0.05, **, *P* < 0.01, ***, *P* < 0.001, and ****, *P* < 0.0001 based on LSD-t test.

Obvious trend changes of TNF-α, IL-6, and IL-1β between the EAE group and EAE_OMAT group were found in the brains and colons at the mRNA levels. We found the mRNA levels of IL-1β (**Figure 8A**), IL-6 (**Figure 8B**), and TNF-α (**Figure 8C**) in the brains of OMAT-treated EAE mice were significantly decreased compared with EAE group mice (all *P<0.05*). Meanwhile, similar phenomena were also observed in the colons with significantly decreased mRNA levels of IL-1β (**Figure 8D**), IL-6 (**Figure 8E**), TNF-α (**Figure 8F**) between the EAE group and EAE_OMAT group (all *P< 0.05*). However, markedly increased mRNA levels of IL-1β (**Figure 8D**), IL-6 (**Figure 8E**), and TNF-α (**Figure 8F**) in the colons were observed in the CON_OMAT group compared with CON group mice (all *P<0.05*), implied that OMAT has certain intestinal toxicity to normal mice.

### 3.9 OMAT improves the function of the BBB and IEB in EAE mice

**Figure 9:**
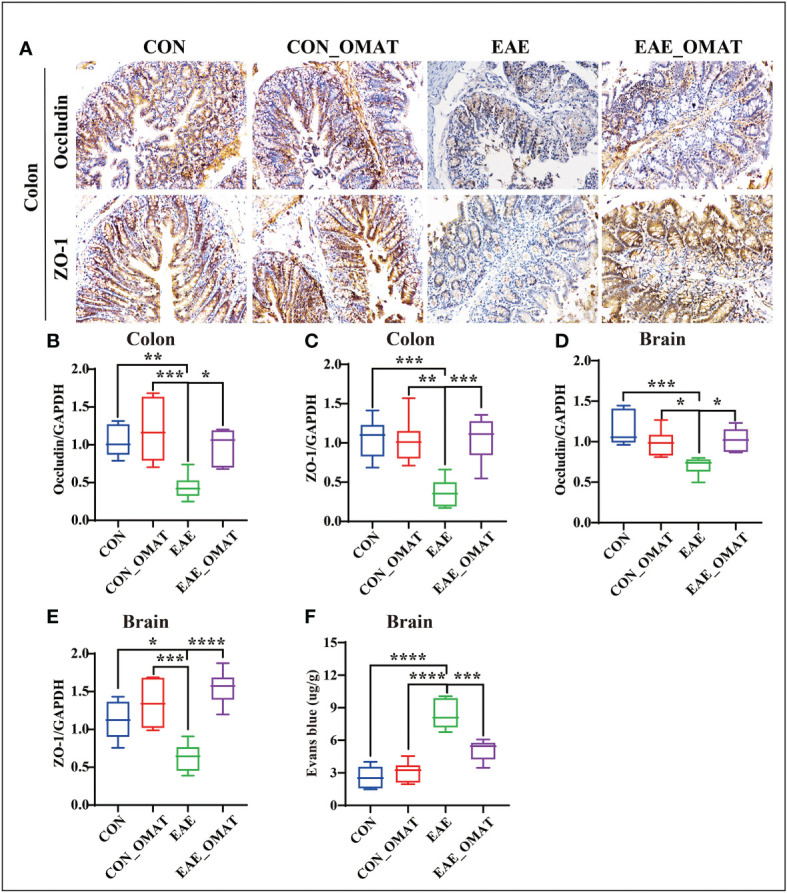
Effect of oxymatrine on the integrity of the blood-brain barrier and intestinal epithelial barrier in EAE mice. **(A)** Representative images of the hippocampus and colon tissue structure were detected by immunohistochemistry staining (n = 6 in each group). Original magnification 20X. **(B, C)** mRNA levels of occludin in the hippocampus **(B)** and colon **(C)**. **(D, E)** mRNA levels of ZO-1 in the hippocampus **(D)** and colon **(E)**. **(F)** Quantitative analysis of Evans blue extravasation in the brain of EAE mice (n = 3 in each group). All data were expressed as the mean ± SD and statistical differences are represented by *, *P* < 0.05, **, *P* < 0.01, ***, *P* < 0.001, and ****, *P* < 0.0001 based on the LSD-t test.

Immunohistochemistry revealed an obvious decrease in the expression of Occludin and ZO-1 in the colons of EAE group mice, while the OMAT reversed this phenomenon (**Figure 9A**). Similar phenomenons for the mRNA levels of Occludin, ZO-1 also occurred in the colons of EAE mice, while reversed by OMAT treatment (all *P<0.05*) (**Figures 9B, C**). The significant change trend of Occludin, ZO-1 mRNA expression in brain tissue is the same as that in colon (all *P<0.05*) (**Figures 9D, E**). The decrease of Occludin and ZO-1 indicated the increased BBB permeability. Quantified the exosmosis of EB dye into the brain as an indicator of BBB permeability, and found EB content in the brain of EAE mice was significantly increased compared with CON mice and CON_OMAT mice, while the phenomenon was prominently reversed by OMAT treatment (all *P* < 0.05) (**Figure 9F**).

## 4 Discussion

The gut microbiome is the largest and most complex microbiome in the human body, which plays an important role in the stability of the intestinal environment and the regulation of the host immune system (Lynch and Pedersen, 2016), and the imbalance of intestinal microbiota structure and function are closely related to a variety of diseases, including MS and other neurodegenerative diseases (Sorboni et al., 2022). This study extends our previous research (Dou et al., 2021) and found OMAT improved the neurological function score in the EAE mice associated with altered alpha-diversity and beta-diversity indexes among the gut microbiomes, hinting that OMAT may play a therapeutic role by regulating the disorder of gut microflora in EAE mice.

Some studies have screened and identified special flora which may be harmful (Clostridium_perfringens, Clostridioides_difficile) to MS, and microbiome transplantation from MS patients has been proven to aggravate the incidence of EAE model mice (Berer et al., 2017; Cekanaviciute et al., 2017). Epsilon toxin from Clostridium perfringens can cross the intestinal wall and BBB by increasing cell permeability and accumulating in the brain, followed by binding the synaptosomal membrane, myelinated structure, glial cells, and oligodendrocytes to lead to demyelination (Linden et al., 2015; Bossu et al., 2020). In the present study, OMAT significantly reduced the content of Clostridium_perfringens in the feces of EAE mice from 3.40% to 0.0025%. The study found that OMAT also significantly reduced the clostridioides_difficile content in feces from 1.33% to 0.00059%. Clostridioides_difficile, possessing an encephalitogenic mimotope of myelin basic protein in the surface layer protein A, could activate autoreactive myelin-specific T cells to induce inflammation infiltration and demyelination in the CNS (Mindur et al., 2020). For common conditional pathogenic bacteria, especially for Escherichia_coli_g:Escherichia-Shigella, OMAT was also significantly reduced by 33.17%, respectively. These findings suggest that OMAT treatment, through modulation of the gut microbiota, can prevent demyelination, which is characteristic of MS.

Studies have also reported that taking probiotics (Lactobacillus, Bifidobacterium, and Streptococcus) or transplanting normal human intestinal flora into EAE model mice can reduce the frequency of inflammatory monocytes CD14^high^CD16^low^ and the expression of HLA-DR on dendritic cells (Tankou et al., 2018; Tankou et al., 2018), and produces myelin antigens to induce an anti-inflammatory peripheral immune response (Kasarello et al., 2015; Kasarełło et al., 2016), regulates the balance of CD4+ T cell subsets (Salehipour et al., 2017), reduces the expression of IL-1β, IL-8, IL-10 and TNF-α in the blood of patients (Tamtaji et al., 2017), and ultimately weaken the severity of the disease (Tamtaji et al., 2017; Mangalam et al., 2017; Tankou et al., 2018; Blais et al., 2021). In the current study, after OMAT treatment, the contents of Lactobacillus and Bifidobacterium, the main components of probiotics in the feces of EAE mice, were increased from 1.25% to 9.82%, and 0.71% to 4.46%, respectively. The abundance of Prevotella histicola in the EAE mice with OMAT treatment was also significantly increased from 0.013% to 1.44%, which was reported could down-regulate the pro-inflammatory Th1/Th17 response and induce regulatory CD4+FoxP3+ regulatory T cells (Treg) to inhibit the occurrence of MS (Mangalam et al., 2017; Blais et al., 2021).

SCFAs, the metabolic products of enteric bacteria, contribute directly to preserving the integrity of the intestinal epithelial barrier (Wan Saudi and Sjöblom, 2017; Feng et al., 2018), and are associated with gut microbiota dysbiosis and inflammation in MS (Zeng et al., 2019; Olsson et al., 2021; Moles et al., 2022). Although the levels of PA and BA in the study increased slightly, while IBA and IVA were dramatically decreased after taking OMAT in EAE mice, further rank regression analysis found the expression of IBA and IVA were significantly positive correlation with intestinal flora disorder, especially among Enterobacter, Escherichia-Shigella, Proteus, Clostridium_innocuum_group, Clostridium_sensu_stricto_1, Clostridioides (all R>0.5). Published data displayed IVA could reduce Na+, K+-ATPase activity (a crucial enzyme responsible for maintaining the basal potential membrane necessary for normal neurotransmission) and enzyme citrate synthase in the cerebral cortex to inhibit the citric acid cycle (Ribeiro et al., 2007; Ribeiro et al., 2009), therefore, the OMAT restores the level of VA may regulate the citric acid cycle by increased the activity of Na+, K+-ATPase and enzyme citrate synthase in EAE mice.

Indeed, alterations of enteric bacteria, will successively activate the intestinal and central immune system, and promote the release of a large number of immune-inflammatory factors, such as IL-1β, IL-6, and TNF-α, which will lead to the change in the permeability of the intestinal mucosal barrier and blood-brain barrier. This view is supported by alterations of intestinal permeability and signs of systemic inflammation in patients with MS which appear to be correlated with the disability status of the disease (Miyauchi et al., 2020; Sorboni et al., 2022). In our study, the relative abundance of the KO05100 pathway (Bacterial invasion of epithelial cells) was significantly increased in EAE mice and reversed by OMAT treatment. Meanwhile, the mRNA expression of IL-1β, IL-6, and TNF-α both in the brains and colons of EAE mice were dramatically increased, and almost completely reversed by OMAT. Consistent with literature reports, the intercellular junction complexes which ensure the integrity of the epithelial barrier and regulate paracellular permeability, including Occludin, ZO-1 (Camara-Lemarroy et al., 2018), located around the apical surface of adjacent epithelial cells, were significantly decreased in EAE mice and reversed by OMAT treatment, and the regulatory effect of OMAT on the IEB or BBB function in EAE model mice was also confirmed in DSS model mice (Yao et al., 2021) or early brain injury rats (Liu et al., 2016).

Surprisingly, the study found OMAT has certain intestinal toxicity to normal mice, but not to EAE mice. Combined with the reported liver protection and liver toxicity, neuroprotection, and neurotoxicity of OMAT in the literature (You et al., 2020; Li et al., 2021), it suggests that the toxic mechanism of OMAT maybe its pharmacodynamic effect in the treatment of MS, hepatitis, and other diseases, which is also in line with an old saying of traditional Chinese medicine “The principle that if there is enough reason, a toxic medicine can also be used without harm”.

**Figure 10:**
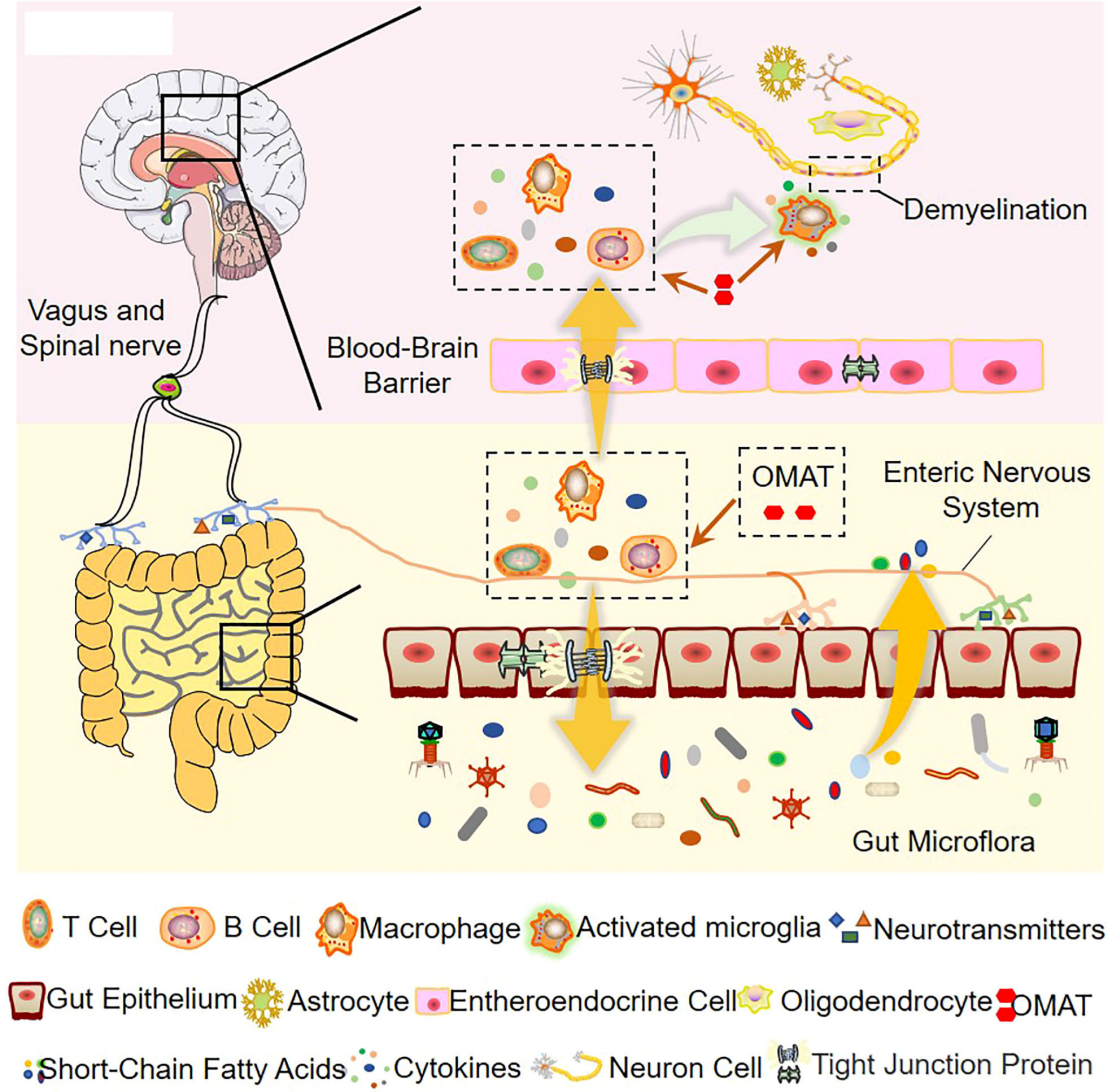
The role of OMAT on interfering with the progression of multiple sclerosis through the brain-gut axis.

Based on the 16S rRNA data, the study concludes that the ecological and functional microenvironment of the gut is differentially altered in the EAE mice and is reversed by OMAT. Collective data indicate that the alterations of the gut microbiome in the OMAT-treated EAE mice may be partly explained by changes in the metabolic pathway of infectious diseases, energy metabolism, immune system, and nervous system. Among the information showed by **Figure 10**, regulating gut microbiota disorder and reducing BBB permeability may be the potential mechanism of OAMT in the treatment of MS.

## Data availability statement

The original contributions presented in the study are included in the article/**Supplementary Material**, further inquiries can be directed to the corresponding author/s.

## Ethics statement

The animal study was reviewed and approved by The First Affiliated Hospital of Henan University of Chinese Medicine (YFYDW2020002).

## Author contributions

J-FT conceived and designed the experiments. M-LZ performed the experiments, analyzed the data, and wrote the manuscript. C-ZW, D-XK, BW, S-QZ, and K-RF were responsible for helping with the collection of experimental samples. Y-LC and X-YW helped to detect the gut microflora and short-chain fatty acids. HZ, L-QY, and LN helped with data analysis. Y-LW and W-XL reviewed the paper. All authors have reviewed the manuscript and approved the final version of the manuscript. All authors have read, revised, and approved the final manuscript.
