# Supplementary material for: Oxymatrine ameliorates experimental autoimmune encephalomyelitis by rebalancing the homeostasis of gut microbiota and reducing blood-brain barrier disruption
Source: Front Cell Infect Microbiol. 2023 Jan 12;12:1095053. doi: 10.3389/fcimb.2022.1095053 (PMC9878311; doi:10.3389/fcimb.2022.1095053)
Supplement: Supplementary file 1 [file Table_1.docx]

**Supplementary Table Legends**

Table S1. Summary of Gene sequence information.

Table S2. Summary of the abundance of microbial species at the phylum level.

Table S3. Summary of the abundance of microbial species at the genus level.

Table S4. Summary of the abundance of microbial species at the species level.

Table S5. Summary of the abundance of microbial species at the phenotype level.

Table S6. Association between the abundance of genus/OTUs microbial and neurological function score.

Table S7. The 16S-based significant changes in abundance for all KOs between any two groups among the four subject groups.

Table S8. Correlation between SCFAs and the genus levels of gut microflora based on *P<0.05* in at least one of the four groups.

Table S1

| **Gene** | **Sequence** | **Length** |
| --- | --- | --- |
| ZO-1 | Forward Primer: GCCGCTAAGAGCACAGCAA | 19 |
|  | Reverse Primer: GCCCTCCTTTTAACACATCAGA | 22 |
| Occludin | Forward Primer: TGAAAGTCCACCTCCTTACAGA | 22 |
|  | Reverse Primer: CCGGATAAAAAGAGTACGCTGG | 22 |
| GAPDH | Forward Primer: AGGTCGGTGTGAACGGATTTG | 21 |
|  | Reverse Primer: GGGGTCGTTGATGGCAACA | 19 |

Table S2

| **Phylum** | **Average relative abundance based on 16S data (%, Mean ± SD)** | | | |
| --- | --- | --- | --- | --- |
|  | **CON** | **CON_OMAT** | **EAE** | **EAE_OMAT** |
| Patescibacteria | 0.17 ± 0.23 | 0.46 ± 0.52 | 0 | 0.02 ± 0.04 |
| Deferribacterota | 0.02 ± 0.02 | 0.01 ± 0.01 | 0 | 0.41 ± 0.79 |
| Proteobacteria | 1.35 ± 1.60 | 0.67 ± 0.27 | 50.03 ± 20.57 | 2.25 ± 2.63 |
| Cyanobacteria | 0.13 ± 0.23 | 0.05 ± 0.04 | 0.01 ± 0.01 | 0.16 ± 0.19 |
| Actinobacteriota | 3.44 ± 1.69 | 5.53 ± 2.26 | 1.75 ± 1.44 | 6.77 ± 4.42 |
| Bacteroidota | 48.16 ± 19.48 | 53.80 ± 12.99 | 19.55 ± 20.17 | 41.27 ± 6.51 |

Table S3

| **Genus** | **Average relative abundance based on 16S data (%, Mean ± SD)** | | | |
| --- | --- | --- | --- | --- |
|  | **CON** | **CON_OMAT** | **EAE** | **EAE_OMAT** |
| Clostridium_innocuum_group | 0 | 0 | 0.92 ± 1.74 | 0.01 ± 0.01 |
| Enterobacter | 0.13 ± 0.12 | 0.01 ± 0.01 | 9.67 ± 8.85 | 0.80 ± 1.12 |
| Proteus | 0 | 0 | 2.37 ± 3.05 | 0 |
| Escherichia-Shigella | 0.38 ±0.57 | 0.01 ± 0.01 | 33.96 ± 19.04 | 0.79 ± 1.43 |
| norank_f__Muribaculaceae | 40.54 ± 14.57 | 44.41 ± 11.68 | 2.97 ± 3.695 | 24.05 ± 7.04 |
| norank_f__Desulfovibrionaceae | 0 | 0.01 ± 0.03 | 0 | 0.61 ± 0.66 |
| Enterococcus | 0 | 0 | 3.97 ± 5.15 | 0.06 ± 0.10 |
| Muribaculum | 1.36 ± 1.58 | 0.42 ± 0.17 | 0.09 ± 0.19 | 0.25 ± 0.25 |
| Dubosiella | 4.21 ± 1.97 | 3.40 ± 1.09 | 0.09 ± 0.14 | 2.02 ± 1.72 |
| Clostridioides | 0 | 0 | 1.33 ± 2.15 | 0 |
| Clostridium_sensu_stricto_1 | 1.17 ± 1.48 | 0.02 ± 0.01 | 4.68 ± 4.31 | 1.46 ± 2.03 |
| Ileibacterium | 3.90 ± 4.51 | 1.55 ± 1.47 | 0.01 ± 0.02 | 0.08 ± 0.09 |
| Alistipes | 1.37 ± 2.14 | 1.17 ± 0.26 | 0.12 ± 0.13 | 1.41 ± 0.94 |
| Rikenellaceae_RC9_gut_group | 1.79 ± 3.36 | 1.35 ± 0.52 | 0.01 ± 0.02 | 0.90 ± 0.95 |
| Desulfovibrio | 1.16 ± 2.32 | 0.05 ± 0.06 | 0 | 0.05 ± 0.06 |
| Helicobacter | 0.43 ± 0.46 | 1.06 ± 0.72 | 0.08 ± 0.11 | 1.08 ± 1.66 |
| norank_f__Eubacterium_coprostanoligenes_group | 0.01 ± 0.01 | 0.01 ± 0.02 | 0.32 ± 0.59 | 1.38 ± 1.84 |
| norank_f__norank_o__Clostridia_UCG-014 | 0.67 ± 0.40 | 1.90 ± 1.75 | 0.03 ± 0.08 | 1.16 ± 1.86 |
| Odoribacter | 0.24 ± 0.20 | 0.63 ± 0.78 | 0.03 ± 0.05 | 0.50 ± 0.44 |
| Lactococcus | 0.01 ± 0.01 | 0.04 ± 0.05 | 1.03 ± 1.55 | 0.09 ± 0.11 |
| Bifidobacterium | 0.49 ± 0.55 | 3.36 ± 2.80 | 0.80 ± 0.99 | 5.12 ± 3.74 |
| Prevotellaceae_UCG-001 | 0.52 ± 1.16 | 1.20 ± 0.80 | 0.12 ± 0.22 | 1.46 ± 2.88 |
| Alloprevotella | 0.25 ± 0.29 | 0.20 ± 0.16 | 1.18 ± 2.11 | 3.11 ± 2.61 |
| Allobaculum | 16.62 ± 19.79 | 11.78 ± 9.09 | 1.12 ± 1.58 | 6.83 ± 5.93 |
| Ruminococcus_torques_group | 0 | 0.09 ± 0.21 | 0.48 ± 0.83 | 2.79 ± 5.72 |
| Parabacteroides | 0.34 ± 0.27 | 1.90 ± 1.35 | 0.50 ± 0.42 | 1.22 ± 1.11 |
| Enterorhabdus | 0.85 ± 0.51 | 0.71 ± 0.50 | 0.23 ± 0.19 | 0.45 ± 0.24 |
| Faecalibaculum | 0.50 ± 0.55 | 0.38 ± 0.28 | 0.01 ± 0.01 | 0.42 ± 0.40 |
| norank_f__Lachnospiraceae | 0.30 ± 0.33 | 0.21 ± 0.26 | 0.97 ± 2.19 | 0.82 ± 0.53 |
| Lachnospiraceae_NK4A136_group | 2.34 ± 2.68 | 2.84 ± 5.91 | 0.49 ± 1.17 | 3.18 ± 4.04 |

Table S4

| **Species** | **Average relative abundance based on 16S data (%, Mean ± SD)** | | | |
| --- | --- | --- | --- | --- |
|  | **CON** | **CON_OMAT** | **EAE** | **EAE_OMAT** |
| Firmicutes_bacterium_M10-2 | 0.81 ± 0.56 | 0.53 ± 0.44 | 0 | 0 |
| Clostridium_perfringens_g__Clostridium_sensu_stricto_1 | 0 | 0 | 3.40 ± 3.33 | 0 |
| Proteus_mirabilis | 0 | 0 | 0.83 ± 1.06 | 0 |
| Escherichia_coli_g__Escherichia-Shigella | 0.38 ± 0.57 | 0.01 ± 0.01 | 33.96 ± 19.03 | 0.79 ± 1.43 |
| Bacteroides_vulgatus | 0.02 ± 0.03 | 0.02 ± 0.02 | 7.81 ± 8.86 | 4.09 ± 5.06 |
| Bacteroides_intestinalis_DSM_17393 | 0 | 0 | 0.34 ± 0.49 | 0.14 ± 0.02 |
| Enterococcus_casseliflavus_g__Enterococcus | 0 | 0 | 1.49 ± 0.72 | 0.03 ± 0.05 |
| Enterococcus_faecalis_g__Enterococcus | 0 | 0 | 2.48 ± 4.84 | 0.03 ± 0.05 |
| Mucispirillum_schaedleri | 0.02 ± 0.02 | 0.01 ± 0.01 | 0 | 0.41 ± 0.79 |
| Erysipelatoclostridium_ramosum | 0 | 0 | 0.12 ± 0.12 | 0.01 ± 0.01 |
| Bacteroides_uniformis | 0 | 0 | 0.38 ± 0.93 | 0.15 ± 0.14 |
| Bifidobacterium_choerinum | 0.01 ± 0.01 | 0.04 ± 0.04 | 0.09 ± 0.16 | 0.63 ± 0.85 |
| Clostridioides_difficile_g__Clostridioides | 0 | 0 | 1.33 ± 2.15 | 0 |
| Bacteroides_thetaiotaomicron | 0 | 0 | 1.31 ± 2.64 | 0.03 ± 0.03 |
| Ileibacterium_valens | 3.90 ± 4.51 | 1.55 ± 1.46 | 0.01 ± 0.02 | 0.08 ± 0.09 |
| Lactobacillus_intestinalis | 0.17 ± 0.23 | 0.19 ± 0.24 | 0.01 ± 0.02 | 0 |
| Paeniclostridium_sordellii | 0 | 0 | 0.33 ± 0.38 | 0 |
| Proteus_vulgaris_g__Proteus | 0 | 0 | 1.54 ± 2.14 | 0 |
| Bacteroides_acidifaciens | 0.08 ± 0.06 | 1.61 ± 1.08 | 3.22 ± 4.88 | 0.77 ± 0.82 |
| Staphylococcus_lentus_g__Staphylococcus | 0 | 0 | 0.20 ± 0.50 | 0.04 ± 0.05 |
| Bifidobacterium_animalis | 0.47 ± 0.54 | 3.31 ± 2.76 | 0.71 ± 0.99 | 4.46 ± 3.55 |
| Streptococcus_respiraculi | 0 | 0 | 0.32 ± 0.40 | 0 |
| Staphylococcus_xylosus | 0 | 0.06 ± 0.05 | 0.10 ± 0.24 | 0.10 ± 0.13 |
| Lachnospiraceae_bacterium_DW59 | 0.17 ± 0.37 | 0.02 ± 0.04 | 0.01 ± 0.02 | 0 |
| Burkholderiales_bacterium_YL45 | 0.37 ± 0.17 | 0.08 ± 0.17 | 0.05 ± 0.12 | 0.24 ± 0.25 |
| Lactobacillus_murinus | 2.62 ± 2.18 | 1.11 ± 0.64 | 1.25 ± 0.88 | 9.07 ± 9.82 |
| Campylobacter_jejuni | 0 | 0 | 0.22 ± 0.36 | 0 |
| Clostridium_aldenense | 0.01 ± 0.01 | 0 | 0.10 ± 0.13 | 0.16 ± 0.37 |

Table S5

| **Phenotype** | **Average relative abundance based on 16S data (%, Mean ± SD)** | | | |
| --- | --- | --- | --- | --- |
|  | **CON** | **CON_OMAT** | **EAE** | **EAE_OMAT** |
| Gram_Negative | 26.80 ± 6.89 | 27.91 ± 6.23 | 13.81 ± 5.78 | 17.70 ± 3.08 |
| Potentially_Pathogenic | 0.34 ± 0.22 | 0.26 ± 0.14 | 7.73 ± 4.64 | 0.714 ± 0.80 |
| Stress_Tolerant | 0.35 ± 0.22 | 0.26 ± 0.14 | 7.73 ± 4.64 | 0.72 ± 0.80 |
| Facultatively_Anaerobic | 0.57 ± 0.24 | 0.41 ± 0.19 | 7.98 ± 4.38 | 0.82 ± 0.84 |
| Anaerobic | 33.51 ± 6.55 | 30.51 ± 7.64 | 16.37 ± 9.82 | 27.53 ± 4.36 |
| Aerobic | 4.58 ± 4.31 | 6.70 ± 3.95 | 2.11 ± 1.05 | 6.36 ± 2.27 |

Table S6

| **Species** | **R** | **P value** |
| --- | --- | --- |
| Bacteroides_acidifaciens | 0.23 (-0.20, 0.59) | 0.2698 |
| Bacteroides_intestinalis_DSM_17393 | 0.75 (0.49, 0.89) | <0.0001 |
| Bacteroides_thetaiotaomicron | 0.80 (0.57, 0.91) | <0.0001 |
| Bacteroides_uniformis | 0.42 (0.00, 0.71) | 0.043 |
| Bacteroides_vulgatus | 0.84 (0.66, 0.93) | <0.0001 |
| Bifidobacterium_animalis | -0.10 (-0.49, 0.33) | 0.6401 |
| Bifidobacterium_choerinum | 0.33 (-0.10, 0.65) | 0.1169 |
| Burkholderiales_bacterium_YL45 | -0.55 (-0.78, -0.18) | 0.0054 |
| Campylobacter_jejuni | 0.54 (0.17, 0.78) | 0.006 |
| Clostridioides_difficile_g__Clostridioides | 0.65 (0.33, 0.84) | 0.0005 |
| Clostridium_aldenense | 0.65 (0.33, 0.84) | 0.0006 |
| Clostridium_perfringens_g__Clostridium_sensu_stricto_1 | 0.84 (0.65, 0.93) | <0.0001 |
| Enterococcus_casseliflavus_g__Enterococcus | 0.86 (0.70, 0.94) | <0.0001 |
| Enterococcus_faecalis_g__Enterococcus | 0.81 (0.59, 0.91) | <0.0001 |
| Erysipelatoclostridium_ramosum | 0.82 (0.62, 0.92) | <0.0001 |
| Escherichia_coli_g__Escherichia-Shigella | 0.74 (0.46, 0.88) | <0.0001 |
| Firmicutes_bacterium_M10-2 | -0.88 (-0.95, -0.73) | <0.0001 |
| Ileibacterium_valens | -0.78 (-0.90,- 0.54) | <0.0001 |
| Lachnospiraceae_bacterium_DW59 | -0.57 (-0.80, -0.21) | 0.0034 |
| Lactobacillus_intestinalis | -0.70 (-0.86, -0.40) | 0.0002 |
| Lactobacillus_murinus | 0.02 (-0.40, 0.43) | 0.9415 |
| Mucispirillum_schaedleri | -0.19 (-0.56, 0.25) | 0.3832 |
| Paeniclostridium_sordellii | 0.46 (0.06, 0.74) | 0.023 |
| Proteus_mirabilis | 0.68 (0.37, 0.85) | 0.0002 |
| Proteus_vulgaris_g__Proteus | 0.72 (0.43, 0.87) | <0.0001 |
| Staphylococcus_lentus_g__Staphylococcus | 0.36 (-0.06, 0.68) | 0.0804 |
| Staphylococcus_xylosus | -0.09 (-0.49, 0.33) | 0.6589 |
| Streptococcus_respiraculi | 0.59 (0.23, 0.81) | 0.0025 |

Table S7

| **Group1** | **Group2** | **KOs with significant changes in abundance** | **Significantly enriched KOs in G1 compared with G2** | **Significantly depleted KOs in G1 compared with G2** |
| --- | --- | --- | --- | --- |
| CON_OMAT | CON | 1 | 1 | 0 |
| CON_OMAT | EAE_OMAT | 19 | 11 | 8 |
| CON | EAE_OMAT | 27 | 10 | 17 |
| EAE | CON | 95 | 43 | 52 |
| EAE | CON_OMAT | 102 | 45 | 57 |
| EAE_OMAT | EAE | 97 | 54 | 43 |

Table S8

| **Genus** | **Related coefficient (R)** | | | | | | | |
| --- | --- | --- | --- | --- | --- | --- | --- | --- |
|  | **SFCAs** | **AA** | **PA** | **IBA** | **BA** | **IVA** | **VA** | **HA** |
| Candidatus_Saccharimonas | 0.43 | 0.28 | 0.41 | -0.18 | 0.62 | -0.32 | 0.12 | 0.26 |
| Clostridioides | -0.36 | -0.16 | -0.60 | 0.53 | -0.46 | 0.50 | -0.33 | -0.09 |
| Dubosiella | 0.31 | 0.12 | 0.45 | -0.34 | 0.45 | -0.37 | 0.11 | 0.28 |
| Muribaculum | 0.54 | 0.31 | 0.75 | -0.44 | 0.46 | -0.55 | 0.14 | 0.08 |
| norank_f__Muribaculaceae | 0.38 | 0.34 | 0.47 | -0.63 | 0.41 | -0.70 | -0.17 | 0.08 |
| Parasutterella | -0.43 | -0.18 | -0.43 | 0.18 | -0.47 | 0.26 | -0.62 | -0.07 |
| Alistipes | 0.35 | 0.43 | 0.28 | -0.60 | 0.44 | -0.55 | -0.04 | -0.01 |
| Clostridium_innocuum_group | -0.44 | -0.30 | -0.34 | 0.59 | -0.64 | 0.63 | -0.10 | -0.01 |
| Clostridium_sensu_stricto_1 | -0.30 | -0.39 | -0.39 | 0.61 | -0.33 | 0.69 | 0.24 | 0.15 |
| Enterobacter | -0.16 | -0.15 | -0.14 | 0.53 | -0.40 | 0.56 | 0.06 | -0.04 |
| Escherichia-Shigella | -0.21 | -0.13 | -0.27 | 0.54 | -0.40 | 0.54 | -0.09 | 0.03 |
| Lactobacillus | -0.18 | -0.11 | -0.28 | -0.42 | 0.04 | -0.33 | 0.05 | 0.26 |
| Prevotellaceae_UCG-001 | 0.33 | 0.48 | 0.28 | -0.57 | 0.31 | -0.58 | -0.08 | 0.09 |
| Proteus | -0.45 | -0.31 | -0.33 | 0.56 | -0.65 | 0.60 | -0.18 | -0.01 |
| Rikenellaceae_RC9_gut_group | 0.31 | 0.37 | 0.30 | -0.49 | 0.43 | -0.48 | -0.03 | 0.19 |
| Allobaculum | 0.24 | -0.02 | 0.40 | -0.25 | 0.54 | -0.21 | 0.37 | 0.02 |
| Bacteroides | -0.26 | -0.01 | -0.11 | 0.03 | -0.46 | 0.12 | -0.23 | -0.10 |
| Colidextribacter | 0.27 | 0.13 | 0.21 | -0.19 | 0.45 | -0.18 | 0.56 | 0.11 |
| Desulfovibrio | 0.59 | 0.41 | 0.30 | -0.32 | 0.76 | -0.36 | 0.33 | 0.20 |
| Enterococcus | -0.27 | -0.10 | -0.27 | 0.37 | -0.58 | 0.39 | -0.21 | -0.11 |
| Helicobacter | 0.25 | 0.23 | 0.03 | -0.36 | 0.48 | -0.35 | 0.19 | 0.04 |
| Ileibacterium | 0.40 | 0.19 | 0.40 | -0.09 | 0.65 | -0.16 | 0.29 | 0.06 |
| Lachnospiraceae_NK4A136_group | 0.34 | 0.13 | 0.12 | -0.19 | 0.60 | -0.19 | 0.64 | 0.21 |
| norank_f__norank_o__Clostridia_UCG-014 | 0.20 | 0.10 | 0.16 | -0.32 | 0.48 | -0.32 | 0.17 | 0.09 |
| norank_f__Oscillospiraceae | 0.24 | 0.09 | 0.02 | -0.24 | 0.43 | -0.14 | 0.57 | 0.23 |
| Odoribacter | 0.61 | 0.53 | 0.37 | -0.29 | 0.73 | -0.31 | 0.38 | 0.30 |
